# 4102 Assessment of ivermectin-treated backyard chickens as a novel urban West Nile virus prevention strategy

**DOI:** 10.1017/cts.2020.370

**Published:** 2020-07-29

**Authors:** Karen Holcomb, Chilinh Nguyen, Brian D. Foy, Christopher M. Barker

**Affiliations:** 1 University of California, Davis; 2 Colorado State University

## Abstract

OBJECTIVES/GOALS: We conducted a randomized field trail to evaluate the efficacy and safety of a novel vector control strategy that involves treating urban backyard chickens with ivermectin (IVM), a widely used antiparasitic and mosquitocial drug. The goal was to reduce vector mosquito populations and West Nile virus (WNV) transmission. METHODS/STUDY POPULATION: We placed eight flocks—four treated and four untreated control—of six Lohmann brown chickens (16 month-old) each in backyard coops across Davis, CA and administered IVM in feed daily at treated coops (200 mg IVM/kg feed) for eleven weeks. We monitored entomological indices weekly (i.e. mosquito abundance, WNV infection prevalence, and parity rate) in *Culex* mosquito populations near (10 m) and far (150 m) from each coop location for the peak WNV transmission season (Jul-Sep 2019). We also monitored serum IVM levels in treated chickens and tested for WNV antibodies in all chickens throughout the study. RESULTS/ANTICIPATED RESULTS: Since IVM impacts only mosquitoes that live long enough to take a bloodmeal from a treated chicken, we do not expect to find a marked difference in adult *Culex* abundance between the two treatment arms, but we expect to find a reduction in WNV infection prevalence and a shift in female mosquito age structure towards younger, uninfected individuals at treated coops. We also anticipate seroconversions in treated chickens to occur at lower rates versus untreated control chickens indicating a reduction in WNV transmission intensity at treated coops. We observed no negative health outcomes from the long-term ingestion of IVM by study chickens. A pathological investigation is underway to compare histological findings between treated and untreated chickens. DISCUSSION/SIGNIFICANCE OF IMPACT: IVM provides the potential for targeted mosquito control. Reduced WNV transmission dynamics here is a stepping stone to a commercial WNV control strategy; IVM-treated feed for wild birds for homeowners’ use to combat WNV transmission in their neighborhoods.

